# Not what it seems: analytical validation and label accuracy of commercial CBD oils using HPTLC

**DOI:** 10.1186/s42238-026-00419-7

**Published:** 2026-03-05

**Authors:** Keilor Morales Alfaro, Gabriel Zamora, Manuel Sandoval-Barrantes, Ana Francis Carballo Arce, Jose A. Rodriguez-Corrales

**Affiliations:** 1 Biorefinery Research and Innovation Center (CIIBio), School of Chemistry, Universidad Nacional, Heredia, Heredia 40101 Costa Rica; 2 Centro de Investigaciones Apícolas Tropicales (CINAT), Universidad Nacional, Heredia, Heredia 40104 Costa Rica; 3 Laboratorio de Analisis y Servicios Quimicos (LASEQ), Universidad Nacional, Heredia, Heredia 40101 Costa Rica

**Keywords:** Hemp, HPTLC, Cannabidiol, Phytocannabinoids, Labeling accuracy

## Abstract

The rapid growth of the cannabidiol (CBD) market has raised concerns about product labeling accuracy and quality control. This study aimed to validate a high-performance thin-layer chromatography (HPTLC) method and apply it to analyze CBD content in commercial oils and e-liquids available in Costa Rica. The HPTLC method was validated following guidelines of the International Council for Harmonization of Technical Requirements for Pharmaceuticals for Human Use. Performance parameters assessed included linearity, accuracy, precision, specificity, and limits of detection (LOD), and quantification (LOQ). After validation, the method was used to quantify CBD and screen for other phytocannabinoids such as CBG, CBN, and Δ⁹-THC in 5 commercial oils and 3 e-liquids. The method showed excellent linearity (R² > 0.9998), precision (%CV < 2%), and accuracy (99–102% recovery), while successfully detecting CBD concentrations and qualitatively identifying other cannabinoids. Notably, some commercial samples showed CBD over- and underlabeling, and tentative presence of Δ⁹-THC. These findings reveal inconsistencies between the labeled and actual CBD content in several products, mirroring international trends, and represent the first documented evidence of mislabeling in hemp-based products in Costa Rica. The validated HPTLC method offers a cost-effective tool for regulatory surveillance, enabling preliminary cannabinoid profiling and quantification. Its application can support public health policies and consumer protection through improved product transparency and compliance with Costa Rica’s national hemp regulations.

## Background

The therapeutic and commercial use of hemp (*Cannabis sativa* L). has expanded rapidly worldwide, driven by evolving regulatory frameworks and increasing interest in the pharmacological properties of its bioactive compounds. Among these, cannabidiol (CBD), a non-psychoactive phytocannabinoid, has gained substantial attention for its documented anti-inflammatory, anxiolytic, and antiepileptic effects. As a result, a wide variety of CBD-containing commercial products such as oils, capsules, cosmetics, and vaporizer liquids have proliferated in consumer markets (Lim et al. 2021, Chu et al. 2024, Han et al. 2024, Britch et al. 2020).

Alongside this expansion, several studies have highlighted a lack of standardization and regulatory compliance regarding the actual CBD content in commercial formulations, raising concerns about public health, therapeutic efficacy, and consumer trust. This problem is particularly pronounced in countries where regulatory systems for medical cannabis remain under development or are inconsistently applied (Peace et al. 2016, Bonn-Miller et al. 2017, Zawatsky et al. 2024, Outhous et al. 2024).

In Costa Rica, the recent legalization of hemp for industrial and therapeutic purposes has opened new commercial opportunities. However, the absence of accredited laboratories capable of verifying cannabinoid concentrations has hindered the ability of health authorities to enforce adequate quality control. Thus, the development of validated and accessible analytical methods becomes essential to support both regulatory institutions, consumers, and domestic producers (Goldman et al, 2021, Rica et al. 2022, Government of Costa-Rica 2024).

High-performance analytical techniques, particularly gas (GC) and liquid (LC) chromatography, are widely used for cannabinoid quantification. GC is commonly coupled to flame ionization detection (FID) or mass spectrometry (MS) due to the organic and semi-volatile nature of the cannabinoids, enabling the analysis of beverages, vape liquids, oils and related matrices with limits of detection (LOD) typically on the order of 0.10 µg/mL and as low as 0.01 µg/mL with tandem MS/MS detectors (Gurley et al. 2020, Ibrahim et al. 2018, Poklis et al. 2019, Bakro et al. 2020, Duchateau et al. 2021). In turn, HPLC is frequently coupled to ultraviolet (UV) and MS detectors. Cannabinoids have been analyzed by reverse phase chromatography in a wide variety of matrices, including oils, cosmetic creams, massage oils, body butters, tinctures, and supplements. Reported LD for HPLC-diode array (DAD) methods are comparable to those obtained by GC-FID or GC-MS, whereas HPLC-MS/MS performs similar to GC-MS/MS (Peace et al. 2016, Mazzetti et al. 2020, Madej et al. 2021, Meng et al. 2018).

Nonetheless, these techniques face several challenges. For instance, HPLC methods based on DAD or single wavelength UV detectors exhibit limited selectivity and sensitivity, since UV absorption provides insufficient spectral information for unequivocal cannabinoid identification, while coelution between structurally related cannabinoids may occur. GC requires high operation temperatures that induce decarboxylation of acidic cannabinoids (e.g., TCHA into TCH), thus requiring derivatization pretreatments. While GC- and HPLC-MS techniques are more sensitive, matrix coelution with analytes may generate false positives or suppress ionization (Vella Szijj 2024, Nahar et al. 2020). In addition, isotopically labeled internal standards are costly and not commercially available for all cannabinoids. Furthermore, GC and HPLC require sophisticated instrumentation (with detectors as costly as $100K), rigorous maintenance, and specialized personnel, which pose significant barriers in resource-limited laboratories.

In this context, high-performance thin-layer chromatography (HPTLC) can serve as a cost-effective screening approach, enabling the simultaneous analysis of multiple samples with reduced solvent usage and simplified operation. In addition, post-chromatographic derivatization provides an additional, orthogonal detection dimension, improving visualization and enhancing selectivity for particular analytes (Outhous et al. 2024, Goldman et al. 2021, Stefkov et al. 2022 , Pourseyed Lazarjani et al. 2020, Barhdadi et al. 2023, Manier et al. 2023). Thus, HPTLC may facilitate preliminary market surveillance and support regulatory monitoring where access to advanced instrumentation is limited.

The aim of the present study was to develop and validate an HPTLC method for the quantitative determination of CBD in commercial products available in the Costa Rican market. The method was designed in accordance with international validation guidelines, including International Council for Harmonization of Technical Requirements for Pharmaceuticals for Human Use (ICH) Q2(R1) and AOAC recommendations. Key performance parameters, such as linearity, accuracy, precision, specificity, stability, and limits of detection and quantification (LOD and LOQ), were evaluated. Following validation, the method was applied to the analysis of various commercial samples, including CBD oils and vaporizer liquids, as well as locally produced hemp-derived products. The measured CBD content was compared with the label claims of each product, revealing significant discrepancies in most cases.

In addition, the presence of other phytocannabinoids such as cannabinol (CBN) and cannabigerol (CBG), as well as trace amounts of Δ^9^-tetrahydrocannabinol (Δ^9^-THC), was tentatively detected in certain vaporizer liquids. These findings underscore the need for mandatory verification protocols and stronger regulatory frameworks in emerging cannabis markets, such as Costa Rica.

## Methods

### Materials and reagents

Certified reference materials (CRMs) of Δ⁹-THC (1.0 mg/mL in methanol) and CBD (1.0 mg/mL in methanol) were procured from Supelco. Analytical grade solvents were used for sample preparation and chromatography development, including ethyl acetate (J.T. Baker), dichloromethane (Merck), diethylamine (Merck), methanol (J.T. Baker), n-hexane (Merck), and p-xylene (Merck). HPLC-grade methanol was procured from J.T. Baker. Developing reagent Fast Blue B salt was purchased from Sigma-Aldrich. High-performance thin-layer chromatography (HPTLC) silica gel 60 F₂₅₄ plates, 20 cm × 10 cm and 10 cm × 10 cm, were procured from Merck.

### Sample preparation and standard solutions

Working standard solutions were prepared by double gravimetric dilution of the CBD reference material, following the procedure outlined in CAMAG Application Note A108.1, (Camag 2017) with minor empirical adjustments (change of mobile phase, vide infra, and development increased by 5 min). A working solution was prepared to construct the calibration curve by diluting a reference material (MR#A) with HPLC-grade methanol. The working solution was analyzed in ten replicates against a certified CBD reference standard (MRC CBD, Cerilliant, 1.0 mg/mL in methanol) to verify its concentration and uncertainty, following the recommendations of the EURACHEM guide for evaluating reference materials in analytical method validation (EURACHEM/CITAC n.d). Briefly, an Ishikawa analysis of cause and effect was constructed and the uncertainty included HPTLC quantification, mass-based dilution factors and uncertainty of MRC.

### Instrumentation and chromatographic conditions

High-performance thin-layer chromatography (HPTLC) analysis was performed using a CAMAG system with VisionCATS software. Plates of HPTLC silica gel 60 F_254_ (20 × 10 cm) were used as stationary phase. The sample and standard applications were performed with an Automatic TLC Sampler 4 in 6 mm bands, allowing for up to 15 applications per plate. Chromatographic development was carried out in an Automatic Developing Chamber 2 using a mobile phase consisting of *p*-xylene: n-hexane: diethylamine (25:10:1) (Liu et al. 2020). Post-run derivatization was performed using 2 mL solution of Fast Blue B salt (10 mg of Fast Blue B salt in water: methanol: dichloromethane 2:5:3). The reagent was prepared daily, approximately 10 min before derivatization, following manufacturer’s (CAMAG) recommendations. Derivatization was performed using a CAMAG Derivatizer that nebulized the solution uniformly over the plate for 3–5 min. After complete application, the plates were allowed to react for 2 min at room temperature. The visualization of the chromatogram was conducted in a TLC Visualizer 2. Images were taken under white light, and ultraviolet light (254 nm and 366 nm). Densitometric analyses were performed with a TLC Scanner 4 at 210 nm and 280 nm (both pre- and post-derivatization) (CAMAG and Application Note 2017, Liu et al. 2020).

### Validation parameters and acceptance criteria

Validation was conducted following the guidelines established by the ICH, as well as HPTLC-specialized literature (Renger et al. 2011). Acceptance criteria were defined according to ICH Q2(R1) and AOAC recommendations (AOAC INTERNATIONAL 2016, Ferenczi-Fodor et al. 2001, International Council for Harmonisation 2005, United Nations Office on Drugs and Crime (UNODC) 2010).

Descriptive, correlational, and inferential statistical analyses were conducted to evaluate compliance with the acceptance criteria for each performance parameter. The limit of detection (LOD) and limit of quantification (LOQ) were calculated as, respectively, 3.33 and 10 times the standard deviation of residuals (Sy) divided by the slope (m) of the calibration curve, respectively, as defined by the ICH guidelines 2023. 

The linear range was defined to encompass ± 20% of the expected analyte content in commercial samples. Linearity was assessed using five calibration points prepared from a working solution of CBD (115.4 ng/µL), applied in volumes of 1, 5, 8, 12, and 15 µL, covering a mass range of 115.4 to 1731 ng. This range was selected based on the LOQ (68 ng), the upper limit of the working range (1836 ng), and the recommendation to use whole µL volumes. Data were processed using the VisionCATS software, applying the linear-2 regression model with area integration, as supported by the behavior of the analyte response and existing literature. Acceptance criteria was *R* > 0.998 across the five calibration levels.

Stability of the analyte was assessed both in solution and on the plate. A 2D-TLC analysis was performed using a 10 cm × 10 cm plate with a CBD reference solution (MR#A, 1370 ng/µL) that had been prepared three months prior to analysis. Accuracy was evaluated through recovery studies using a commercial CBD oil sample spiked at three concentration levels (low, medium, and high) within the working range. Spiking was performed by overspotting known amounts of a CBD reference solution onto sample tracks using the same working solutions prepared for the repeatability test (vide infra). On a single plate, three replicate enriched samples were applied per level, along with one replicate of the unspiked sample and a five-point calibration curve. Acceptance criterion was recovery between 95 and 105%.

Specificity was assessed by comparing the retention factor (Rf) of the analyte to nearby signals within ± 25% of the analyte Rf. Precision was evaluated by calculating the coefficient of variation (CV) across different days (intermediate precision) and within the same day (repeatability), using acceptance criteria of %CV < 3% for both parameters. Accuracy was assessed as the percentage recovery of spiked samples at three concentration levels. Uncertainty in the preparation of the working solutions was estimated following EURACHEM/CITAC CG4 guidelines using gravimetric dilution data (Analíticas et al. n.d).

### Application to commercial products

To evaluate the applicability of the validated HPTLC method to commercial CBD oil products, a preliminary market survey was conducted in early 2023. Visits were made to ten pharmacies and ten healthy food stores located in Heredia and Alajuela, Costa Rica, as well as fifteen online stores. The validated method was applied to the analysis of CBD oils and e-liquids, with CBD content determined and compared to product label claims. In addition, presence of other cannabinoids such as cannabinol (CBN), cannabigerol (CBG), and Δ^9^-tetrahydrocannabinol (Δ^9^-THC) was assessed (CAMAG 2017, Liu et al. 2020, U.S. Pharmacopeia (USP) 2022, Yuwono et al. 2005).

Each sample was subjected to six to eight replicate measurements, which were integrated into the specificity and extract characterization tests. The combined expanded uncertainty of the mean CBD concentration was calculated for each product using a coverage factor of k = 2, taking into account key sources of systematic and random error.

Furthermore, cannabinoid content was analyzed in three commercial CBD e-liquids. Sample pretreatment involved double gravimetric dilution with methanol to achieve target concentrations of 0.25 mg/mL (sample 8) and 0.5 mg/mL (samples 6 and 7), corresponding to theoretical CBD loads of 1000 ng per plate spot.

## Results

### Performance parameter evaluation

The HPTLC method was validated against established performance parameters and acceptance criteria, as discussed below.

#### Stability

**Fig. 1:**
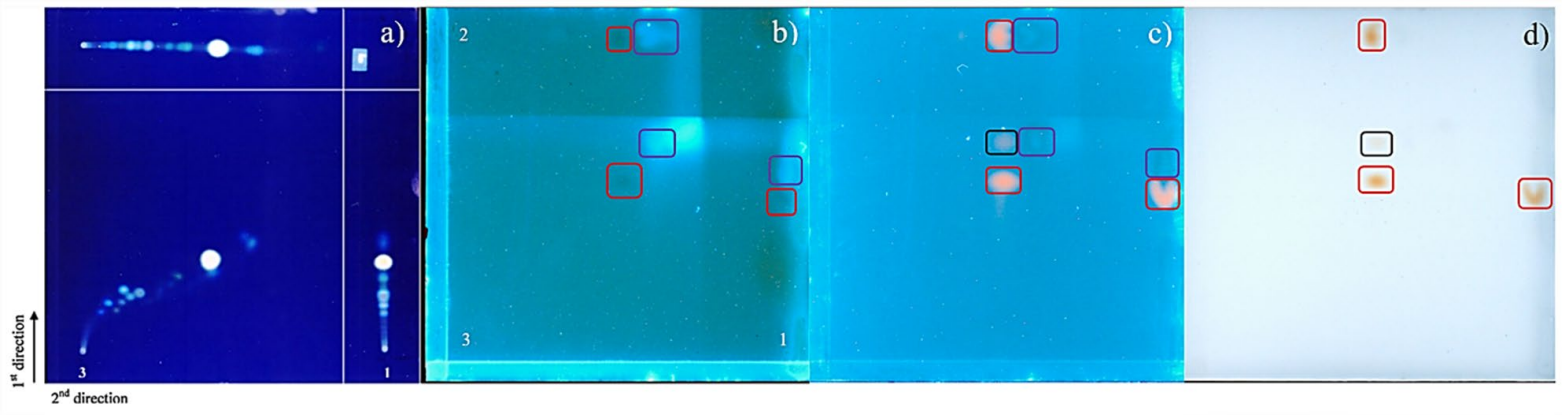
Comparison of images acquired during the evaluation of the stability parameter using 2D-TLC. **a** example of analyte degradation (Renger et al. 2011), reprinted from Journal of Chromatography A, Vol. 1218, B. Renger, Z. Végh, K. Ferenczi-Fodor, Validation of thin layer and high-performance thin layer chromatographic methods, pp. 2712–2721, Copyright (2011), with permission from Elsevier, (**b**) developed plate visualized at 366 nm, (**c**) derivatized plate visualized at 366 nm, (**d**) derivatized plate visualized under white light. Red boxes indicate CBD bands, purple boxes highlight medium chain triglyceride oil from CRM#A migrating with the solvent front, and black boxes show solvent front region, potentially containing an excess of applied analyte

A 2D-TLC test was conducted using a 10 × 10 cm TLC silica gel 60 F₂₅₄ plate (see Fig. 1). A CBD reference solution (MR#A) was applied in three positions, and the plate was developed in two perpendicular directions (first along the Y-axis, and then along the X-axis) with mobile phase used for HPTLC (*vide supra*). Since CBD is not directly visible under 366 nm light, the plate was derivatized with Fast Blue B salt—a chromogenic reagent for cannabinoids—allowing visualization under UV and white light.

As displayed in Fig. 1, no evidence of degradation products or anomalous spots beyond those attributed to CBD were observed post-derivatization. In contrast, an example image of a degraded analyte included in the study (Fig. 1a) demonstrated signal dispersion in both axes, which was consistent with breakdown. The absence of such patterns in the tested CBD reference confirmed its stability. No significant variation was observed in the analytical response, and no visual evidence of analyte degradation was detected in the stored reference material, confirming the stability of the analyte under the evaluated conditions. These observations align with literature reports indicating that cannabinoid solutions remain stable for up to 12 months when stored below 5 °C and protected from light—conditions that were maintained throughout this study (Franco et al. 2022).

#### Limit of detection and limit of quantification

Based on data from the initial specificity test (S = 9.94 × 10⁻⁶ AU/ng; Sy = 6.77 × 10⁻⁵ AU), the LOD was estimated at 22 ng and the LOQ at 68 ng. To experimentally verify the LOD, a CBD solution at 11.6 ng/µL was applied in duplicates from 1 to 8 µL (11.6–92.8 ng), alongside matrix blanks prepared from diluted medium-chain triglycerides (MCT) oil. At 23.2 ng, the analyte signal was clearly distinguishable from the noise, whereas at 11.6 ng, the signal resembled that of the blank. These findings confirmed the calculated LOD value.

#### Working range

**Fig. 2:**
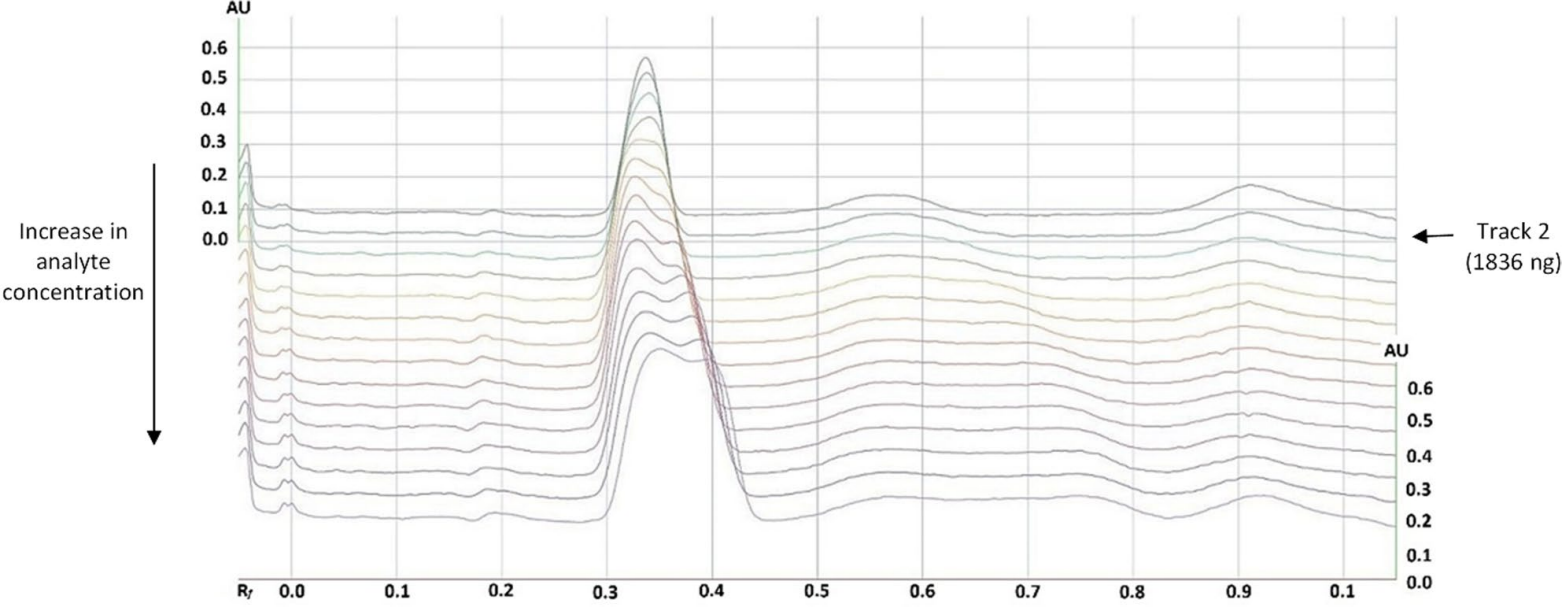
Comparison of chromatograms acquired at 210 nm during the evaluation of the upper limit of the working range

The working range was established based on ICH Q2(R1) guidelines, which recommend covering at least ± 20% of the expected analyte concentration to ensure acceptable linearity, accuracy, and precision in the analysis of finished products. To determine the upper limit of the working range, a high-concentration CBD solution (1147.8 ng/µL) was applied in volumes corresponding to 1492–5280 ng. As shown in Fig. 2, analyte signals above 1836 ng began to lose their Gaussian shape, resulting in signal broadening and splitting—an effect attributed to overloading the stationary phase, causing excess analyte to migrate with the mobile phase without interacting with the saturated stationary phase. Based on this observation, the working range was capped at 1836 ng per track. Although the LOQ was experimentally determined at 68 ng, the lowest calibration level was set at 115.4 ng to ensure conservative quantification within the calibration range.

Subsequent evaluation of linearity, precision, and accuracy confirmed that the selected calibration curve range (115–1731 ng) encompassed at least ± 20% of the expected analyte content, ensuring the suitability of the method for routine analysis of CBD concentrations across typical commercial formulations.

#### Linearity

Four parameters were used to evaluate linearity: (1) correlation coefficient (R), (2) variation coefficient of the calibration function (%CV CAMAG), (3) relative standard deviation of the process (VXO), and (4) visual analysis of residuals. Across five calibration curves, R values ranged from 0.99989 to 1.00000, with all %CV CAMAG values < 1.2% and VXO values < 1.3%, well below the 5% threshold (Yuwono et al. 2005, Srivastava 2011). In all cases, residuals showed random distribution around zero without any discernible trend, confirming excellent fit to the linear model (Table 1). These results demonstrate a high degree of linearity, precision, and consistency in the analyte response across the tested range, supporting the suitability of the method for quantitative CBD analysis.

**Table 1:** Linearity parameters of the HPTLC method for quantitative CBD determination

Calibration curve	*R*	*R*²	Slope (AU/ng)	Intercept (AU)	CV CAMAG (%)	Vxo (%)	Residual trend
1	0.9999	0.9999	1.23 × 10⁻³	1.30 × 10⁻⁵	0.64	0.90	No
2	0.9999	0.9998	7.69 × 10⁻⁶	1.39 × 10⁻⁵	1.14	1.17	No
3	1.0000	0.9999	9.94 × 10⁻⁶	1.92 × 10⁻³	0.52	0.74	No
4	0.9999	0.9998	9.26 × 10⁻⁴	1.23 × 10⁻⁵	1.03	1.24	No
5	0.9999	0.9999	8.26 × 10⁻⁴	1.12 × 10⁻⁵	0.67	0.96	No
Average	0.9999	0.9998	5.99 × 10⁻⁴	3.95 × 10⁻⁴	0.8	1.00	No

#### Specificity

Raw chromatograms acquired at 210 nm were analyzed to evaluate the method’s specificity. Retention factors (Rf) were extracted for all detected signals from both sample and reference tracks. Signals not corresponding to CBD within ± 25% of the CBD reference Rf value were assessed for potential interference. Three groups were defined: substances that eluted before the reference (− 25%, Rf 0.2656–0.3542), the reference itself (Rf 0.3542), and substances that eluted after the reference (+ 25%, Rf 0.3542–0.4428). For each group, the mean Rf and standard deviation were calculated to establish their respective Rf ranges (mean ± SD). After analysis of all commercial samples, only one signal was detected in the range prior to the reference (Rf = 0.2817 ± 0.0098, *n* = 6) and one was detected after the reference (Rf = 0.433 ± 0.021, *n* = 3).

Furthermore, HPTLC-specialized validation literature (Liu et al. 2020) suggests assuming a ± 10% variation in the Rf for potential interferences as an outermost scenario, thus yielding ranges of 0.2535–0.3098 and 0.3900-0.4767 for the two potential interferences identified. The reference signal (Rf 0.3542 ± 0.0062; range 0.3480–0.3604) did not overlap with the average or outermost Rf ranges of these nearby signals, confirming that the method can clearly distinguish CBD from adjacent matrix components and, thus, satisfies the specificity criterion.

#### Intermediate precision and repeatability

For intermediate precision, the %CV ranged from 0.90% to 1.6%, whereas for repeatability it ranged from 0.13% to 0.45%. All values were below the predefined acceptance criteria of %CV < 3% for intermediate precision and %CV < 2% for repeatability, confirming the method’s high degree of precision (Table 2) (Renger et al. 2011, Srivastava 2011).

**Table 2:** Validation parameters of HPTLC methods for cannabinoids: repeatability and intermediate precision

Matrix analyzed in the study	Quantification range	Linearity (*R*²)	Repeatability (% CV)	Intermediate precision (% CV)	Reference
Cannabis plant material	0.50–9.5 µg	0.9996	> 5.7	> 6.0	^Duffau and Alcamán 2019^
Cannabis-derived products, plant material	25–500 ng	0.9943	–	–	^Liu et al. 2020^
Oils enriched with hemp extract	115–1731 ng	0.9998	0.13–0.45	0.9–1.6	Present study

#### Accuracy

The recovery percentages ranged from 99.46% to 101.25% across the three levels, with %CV values below 1%. These results demonstrate the method’s high accuracy, as all recoveries fall within the predefined acceptance range of 95–105%.

#### Overall assessment

The HPTLC method was successfully validated, showing reliable performance across all assessed parameters. The analyte showed no signs of degradation, confirming its stability under the tested conditions. The method exhibited a low LOD (22 ng) and LOQ (68 ng), with a validated working range of 115.4–1730.7 ng, exceeding the minimum recommended scope while maintaining analytical robustness. Linearity was excellent (*R* ≥ 0.99989), with minimal variation and no residual trends. The method showed high specificity, precision (%CV < 1.6%), and accuracy (99–101% recovery), supporting its suitability for the quantification of CBD in commercial formulations.

### Analysis of CBD in commercial hemp-enriched oils

**Fig. 3:**
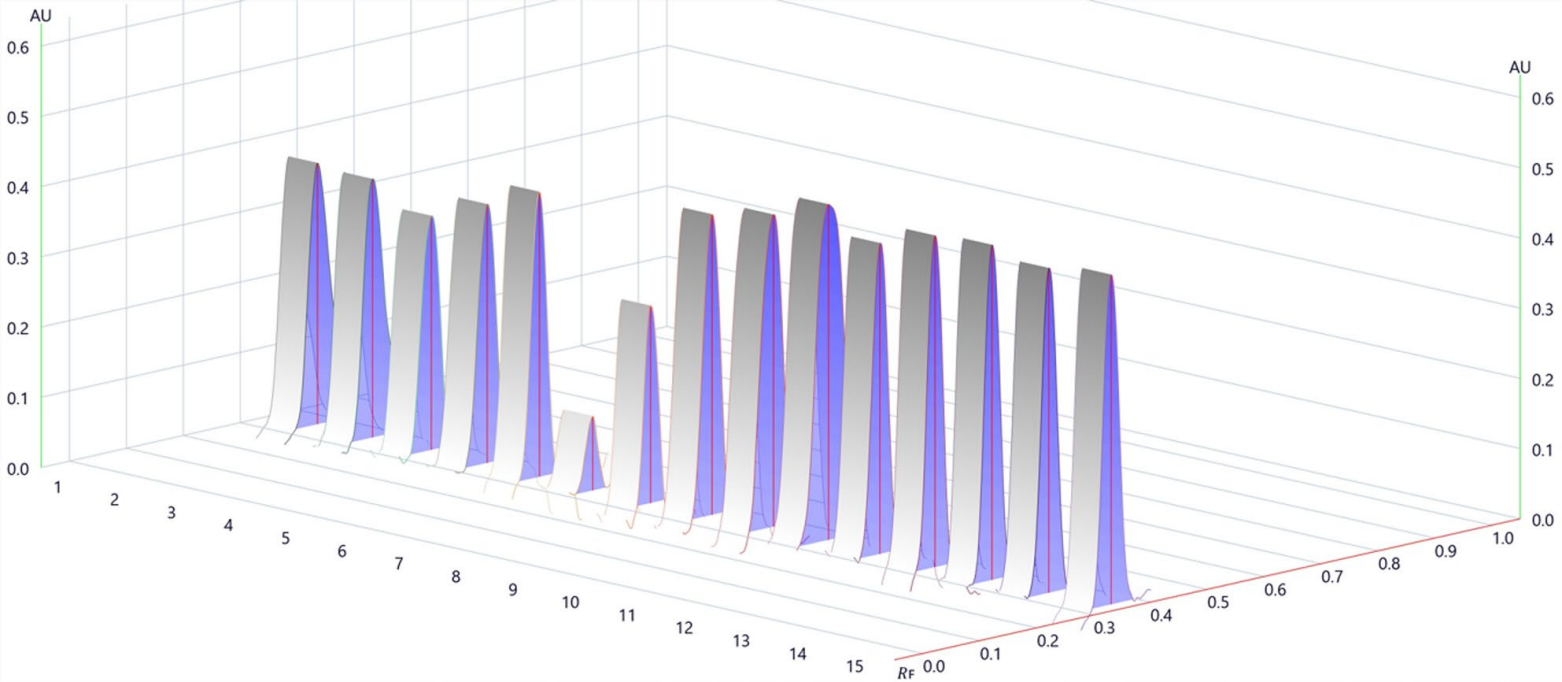
Integrated chromatograms acquired at 210 nm during the quantification of CBD content in commercial hemp-enriched oil samples using the visionCATS software. Commercial samples (1–5, 11–15), calibration curve (6–10)

The validated HPTLC method was applied to five commercial CBD oil samples to determine their actual cannabidiol content and evaluate labeling accuracy. CBD content was determined by interpolating the integrated chromatographic signals (210 nm) against a five-point calibration curve applied on the same plate (see Fig. 3).

Table 3 summarizes key information on the products, including country of manufacture, declared CBD content, the measured average concentrations with expanded uncertainty (U, k = 2), the ratio between experimental and labeled values expressed as a percentage, and the coefficient of variation (%CV) for each determination. The samples included products formulated with hemp extracts of various origins, primarily from the United States. The results revealed significant discrepancies in some cases, with one product exceeding the labeled content by over 39% and others falling below the 90% threshold typically recommended for labeling accuracy.

**Table 3:** Origin data and results obtained for the determination of CBD concentration in commercial hemp-enriched oil samples

Commercial sample	1	2	3	4	5
Hemp origin in sample	Not available	Oregon, USA	Oregon, USA	Colorado, USA	Not available
Theoretical value (mg/mL)	8.8	10	28	50	156
Average concentration ± U (k = 2) (mg/mL)	12.25 ± 0.99	8.86 ± 0.73	25.0 ± 2.2	51.2 ± 4.4	161 ± 15
Ratio experimental result/label (%)	139.2	88.6	89.3	102.5	103.4
% CV in determination	0.45	0.88	0.99	1.6	2.3

### Characterization of additional phytocannabinoids in commercial hemp-enriched oils

**Fig. 4:**
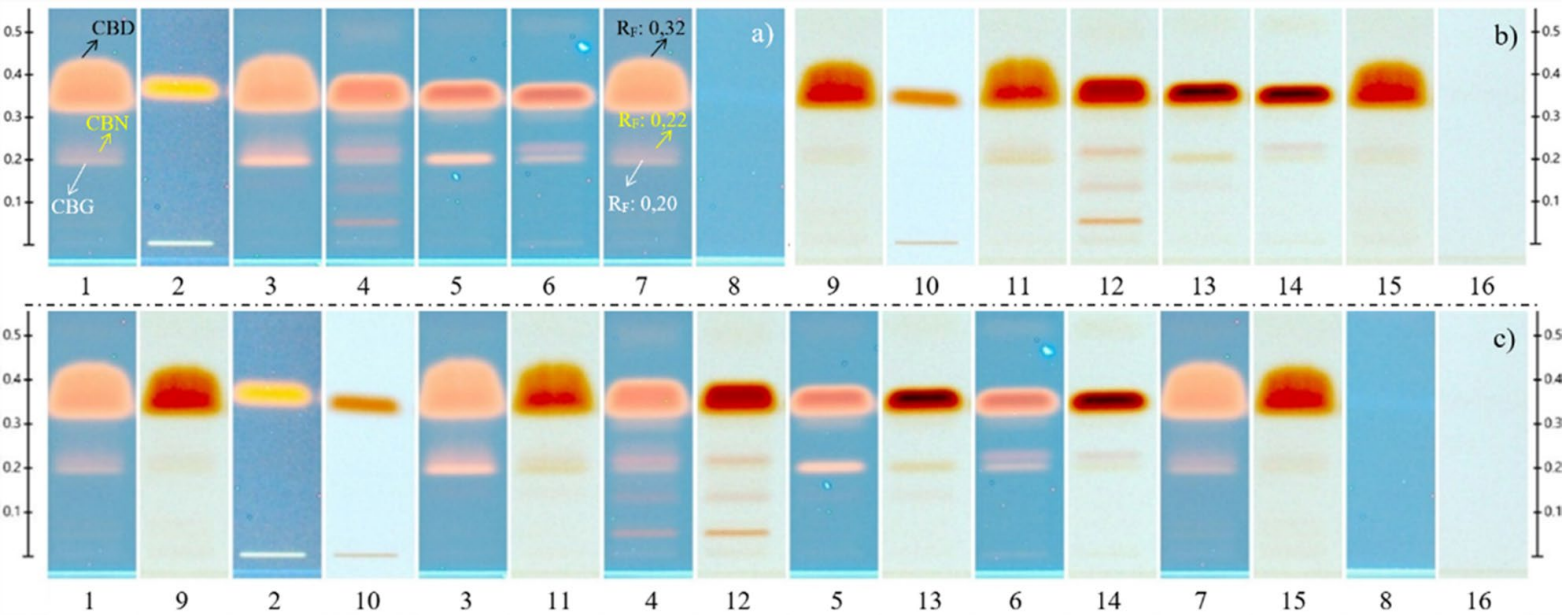
Characterization of the analyzed commercial samples: (**a**) visualization of the derivatized plate at 366 nm, (**b**) visualization of the derivatized plate under white light, and (**c**) image comparison. Cannabinoid reference material (1, 7, 9, 15), sample 1 (2, 10), sample 2 (3, 11), sample 3 (4, 12), sample 4 (5, 13), sample 5 (6, 14), matrix blank (8, 16)

During the quantification of CBD in commercial hemp-enriched oil samples, additional chromatographic signals were observed after plate derivatization with Fast Blue B salt. Given that the CBD reference material (MR#A) also contained CBN and CBG, a qualitative test was conducted with higher sample volumes to identify these additional cannabinoids. As shown in Fig. 10, visual comparisons of derivatized plates under 366 nm UV light and white light revealed bands corresponding to CBN and CBG, identified based on their retention factors (Rf) and color response relative to the reference (see Fig. 4).

Qualitative analysis indicated that Samples 3 and 5 contained CBD, CBN, and CBG, whereas samples 2 and 4 contained only CBD and CBG. Sample 1 showed no evidence of cannabinoids other than CBD. The R_f_ values are consistent with those of published HPTLC characterization protocols and the CAMAG application note used as a reference (CAMAG and Application Note 2017, Liu et al. 2020).

### Analysis of CBD e-Liquids for vaping devices

Three commercial CBD e-liquids were analyzed to evaluate their cannabinoid content. Two of the three products presented lower-than-labeled CBD contents, particularly sample 6 (3.5 mg/mL vs. 16.7 mg/mL on label, with overlabeling of 13,2 mg/mL or 79,0%).

After derivatization with Fast Blue B salt, an unexpected chromatographic signal was detected in the samples, which was distinct from those of CBD, CBG, and CBN. Based on previously reported Rf and coloration (Sherma 2019), the signal was preliminarily identified as Δ⁹-THC. Although product degradation and analyte transformation were hypothesized as potential sources of false positives, literature evidence indicates that CBD degradation into Δ⁹-THC requires highly specific conditions—such as elevated temperatures, strongly acidic environments, or prolonged light exposure—which were not present during storage. A second experiment using a certified Δ⁹-THC reference standard (53.6 ng/µL) confirmed this identity by comparison of retention factors and color response.

**Fig. 5:**
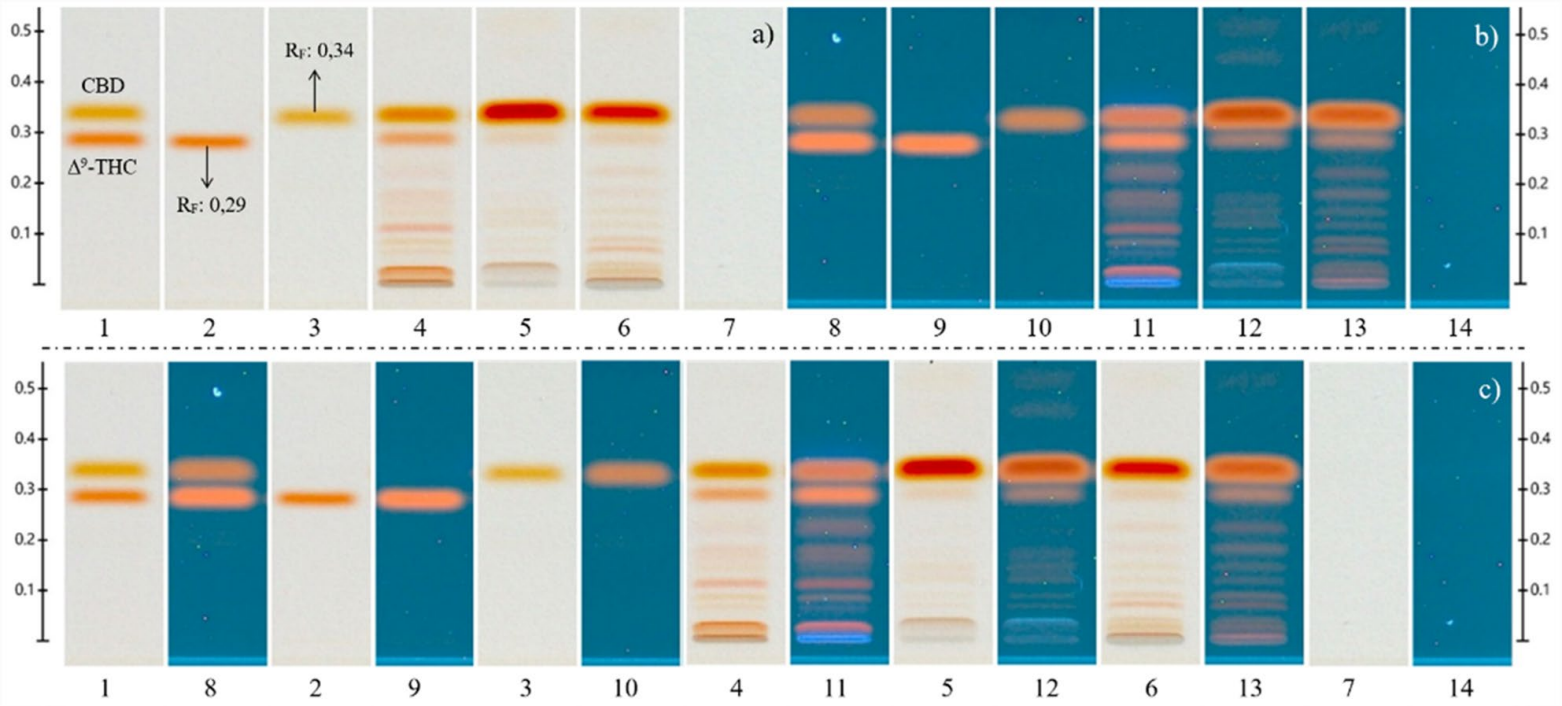
Analysis of CBD-containing e-liquids for vaporizers and electronic cigarettes: (**a**) visualization of the derivatized plate under white light, (**b**) visualization of the derivatized plate at 366 nm, (c) image comparison. Reference overspot (1, 8), Δ⁹-THC reference (2, 9), CBD reference (3, 10), sample 6 (4, 11), sample 7 (5, 12), sample 8 (6, 13), and methanol solvent blank (7, 14)

## Discussion

The successful validation of the HPTLC method for CBD quantification offers a pragmatic balance between performance, throughput, and reliability. This study demonstrated that the technique can reliably deliver key validation parameters such as linearity, precision, and specificity, in compliance with international guidelines. These findings support the adoption of HPTLC not only in academic and research contexts, but also by producers and regulatory agencies.

### Implications of label discrepancies on public health and consumer trust

The observed deviations between labeled and measured CBD concentrations in several commercial products are not trivial. Under- or over-labeling can lead to subtherapeutic dosing, economic deception, or unintended pharmacological effects. Such inconsistencies may also erode consumer trust in a rapidly growing industry. By highlighting these gaps, the present study highlights the urgent need for consistent analytical oversight and product certification, especially in countries where regulatory systems are still maturing. Notably, it represents the first assessment of CBD content in hemp-derived commercial products in Costa Rica, providing an early snapshot of a market that has emerged over the past decade in this Latin American country.

A growing body of international literature reveals widespread inconsistencies between labeled and actual CBD content in commercial oils, stressing the global issue of product mislabeling. In the United States, two independent studies reported accurate labeling in only 36.4% and 28.6% of samples, respectively, with a considerable percentage either under-labeled or over-labeled, and some products showing deviations as high as 53% above the stated CBD content (Miller et al. 2022, Gidal et al. 2024). Similarly, in the United Kingdom (Liebling et al. 2022) and Hungary (Vida et al. 2023), appropriate labeling was confirmed in just 37.9% and 50% of samples, respectively, with under- and over-labeling remaining significant (Liebling et al. 2022, Vida et al. 2023). The findings of the present study in Costa Rica align with these international trends, with only 20% of the tested oils falling within the acceptable 90–110% labeling range and 70% of products being over-labeled.

These discrepancies raise concerns about quality control practices in the CBD industry, both locally and globally. Inaccurate labeling can result in consumers receiving less CBD than expected—or, conversely, ingesting doses above intended levels—which may have economic, regulatory, and safety implications. These results emphasize the urgent need for regulatory oversight and routine analytical verification of cannabinoid content in commercial products, both to protect consumers and to foster trust in the emerging CBD market.

### Role of HPTLC in strengthening national surveillance capacity

The validation and application of the HPTLC method developed in this study gains significant importance considering the recent Costa Rican Technical Regulation RTCR 511:2023, which establishes the administrative provisions, labeling requirements, analytical specifications, and sanitary controls for health-related products containing hemp (*Cannabis sativa* L.). This decree regulates foods, dietary supplements, cosmetics, and other non-psychoactive hemp-derived products, setting clear and enforceable limits for phytocannabinoid content—primarily CBD and THC.

Among the most relevant provisions is the requirement that cannabinoid content be stated on product labels in concentration units or per serving amount, with an acceptable deviation of no more than ± 15% from the analyzed value. Furthermore, the regulation defines maximum daily intake thresholds for dietary supplements (70 mg/day of CBD and 0.42 mg/day of THC) and processed foods, depending on the product type. To ensure compliance, the decree mandates that each marketed batch undergo laboratory testing by facilities accredited under INTE-ISO/IEC 17,025, using reference techniques such as HPLC or other validated instrumental methods.

The proposed HPTLC method offers a cost-effective and reliable tool for preliminary screening of CBD content, complementing official reference methods. The successful detection of labeling discrepancies in this study underscores its utility for routine inspections and regulatory surveillance, particularly in resource-limited settings. This method could support public health protection, commercial transparency, and the enforcement of hemp product regulations in Costa Rica by strengthening national analytical capacities.

### Detection of additional phytocannabinoids and labeling discrepancies

Qualitative detection of additional phytocannabinoids, such as CBG and CBN, revealed discrepancies between product labels and actual contents, highlighting the need for clearer classification standards and analytical verification. While Samples 2–4 matched their broad-spectrum labeling, Sample 1, labeled as “hemp extract,” lacked other cannabinoids and resembled a CBD isolate. These findings underscore the value of validated methods for confirming cannabinoid profiles to support accurate product classification and regulatory oversight.

The tentative detection of Δ⁹-THC in e-liquids raises concerns regarding either adulteration or misformulation and mirrors previous reports from New York and Mississippi, USA (Gurley et al. 2020, Duffy et al. 2020). Although current regulatory frameworks often focus on THC limits in edibles and tinctures, vaping products remain comparatively underregulated. This highlights the need to extend analytical verification and compliance requirements to vaporizer liquids, especially considering the heightened bioavailability of cannabinoids through inhalation routes and the potential for psychoactive exposure. A limitation of the present study is that THC identification was based on HPTLC R_f_ matching and comparison with certified standards. While this approach is suitable for preliminary screening and has been reported for cannabinoid profiling, definitive structural confirmation would benefit from GC-MS or LC-MS/MS. The absence of such instrumentation during the study limited confirmatory analysis. Furthermore, the results reflect products available during the defined 2023 sampling period and should not be interpreted as representative of subsequent market conditions.

## Conclusions

The HPTLC method validated in this study demonstrated excellent analytical performance across all evaluated parameters, including linearity, precision, accuracy, specificity, and analyte stability. Its ability to reliably quantify CBD within a broad concentration range confirms its suitability for routine analysis of commercial hemp-derived products.

Application of the method to commercial CBD oils and e-liquids available in Costa Rica in 2023 revealed widespread inconsistencies between labeled and actual CBD content. Only 20% of tested products met acceptable labeling accuracy standards (90–110%), with 70% of the samples exceeding their declared content, raising concerns about regulatory compliance and consumer safety. These findings are consistent with international reports, underscoring a global trend of mislabeling in the CBD product industry, and provide the first baseline assessment of labeling accuracy in the Costa Rican market.

In addition to CBD quantification, this method allowed the qualitative identification of other phytocannabinoids, such as CBG, CBN, and Δ⁹-THC. The tentative detection of Δ⁹-THC in certain vaping products suggests possible adulteration, poor purification, or intentional inclusion, further highlighting the need for regulatory oversight and product testing across all cannabis-derived formulations.

The ability of this HPTLC method to detect and differentiate key cannabinoids makes it a valuable tool for preliminary market surveillance and the enforcement of quality standards. It complements officially mandated reference methods and can be implemented in national laboratories to support the requirements established by Costa Rica’s RTCR 511:2023 technical regulation.

Ultimately, the application of a cost-effective and validated analytical method, such as the HPTLC method presented here, can strengthen public health protection, fostering transparency in the growing CBD industry, and supporting the successful implementation of hemp product legislation in emerging markets. Within the 2023 sampling campaign, this study establishes a baseline assessment of labeling accuracy and cannabinoid presence in commercially available hemp-derived products. Broader evaluations incorporating larger and temporally updated sampling sets, expanded product categories, and confirmatory analytical techniques would further strengthen understanding of market compliance in both emerging and established hemp-product markets.

## Data Availability

The chromatograms generated during the current study are available from the corresponding author upon reasonable request.

## Abbreviations

CBD: Cannabidiol
HPTLC: High-performance thin-layer chromatography
CBG: Cannabigerol
CBN: Cannabinol
Δ⁹-THC: Δ^9^-tetrahydrocannabinol
ICH: International Council for Harmonization of Technical Requirements for Pharmaceuticals for Human Use
LOD: Limit of detection
LOQ: Limit of quantification
HPLC: High-performance liquid chromatography
GC-MS: Gas chromatography coupled to mass spectrometry
LC-MS/MS: Liquid chromatography coupled to mass spectrometry/mass spectrometry
CRM: Certified reference material
TLC: Thin-layer chromatography
CV: Coefficient of variation
MCT: Medium-chain triglycerides
VXO: Relative standard deviation of the process
AU: Arbitrary units
